# Multiple contaminant detection in commercially available chicken-based cat kibbles in Italy

**DOI:** 10.3389/fvets.2026.1846767

**Published:** 2026-06-10

**Authors:** Yasin Öztürk, Mario Nicotra, Francesco Alessandro Palermo, Carlotta Marini, Merve Öztürk, Roberta Tardugno, Natalia Pedretti, Andrea Marchegiani, Alfredo Di Cerbo, Paolo Cocci, Serena Gabrielli, Marco Minicucci, Anisa Bardhi, Andrea Barbarossa, Alessandro Di Cerbo

**Affiliations:** 1 Department of Pharmacology and Toxicology, Faculty of Veterinary Medicine, University of Necmettin Erbakan, Ereğli, Konya, Türkiye; 2 School of Biosciences and Veterinary Medicine, University of Camerino, Matelica, Italy; 3 Department of Internal Medicine, Faculty of Veterinary Medicine, University of Necmettin Erbakan, Ereğli, Konya, Türkiye; 4 Department of Pharmacy-Drug Sciences, University of Bari “Aldo Moro,” Bari, Italy; 5 Hip-Tech PhD Program, University of Modena and Reggio Emilia, Modena, Italy; 6 Varano-Antinori High School, Camerino, Italy; 7 School of Science and Technology, Chemistry Division, ChIP Research Center, University of Camerino, Camerino, Italy; 8 School of Science and Technology, Physics Division, University of Camerino, Camerino, Italy; 9 Department of Veterinary Medical Sciences, University of Bologna, Ozzano dell'Emilia, Bologna, Italy

**Keywords:** antibiotic residues, bisphenol A, chicken-based pet food, microplastics, perfluorinated and polyfluorinated alkyl substances, toxic metals

## Abstract

The increasing consumption of commercially available pet food raises growing concerns regarding the possible presence of contaminants and their potential impact on companion animal health. In this sense, pet food, particularly chicken-based, may become the main vehicle for environmental and anthropogenic pollutants and the underlying cause of most inflammatory pathologies. This research aimed to investigate the possible presence of contaminants or their residues in 29 commercially available chicken-based cat kibbles using Liquid Chromatography-Tandem Mass Spectrometry (LC-MS/MS) for antibiotics, per-and polyfluoroalkyl substances (PFAS), Inductively Coupled-Plasma Mass Spectrometry (ICP-MS) for toxic metals, and Enzyme-Linked Immunosorbent Assay (ELISA) for bisphenol A (BPA). Microplastics (MPs) were determined using sodium chloride (NaCl) density separation extraction, followed by repeated recovery steps, on-filter oxidative digestion, and microscopic inspection. Chlortetracycline residues were present only in 1 out 29 samples (79 ± 35 μg/kg), doxycycline residues were detected in 4 out of 29 samples (mean concentration 80.25 ± 35.5 μg/kg), polyfluoroalkyl substances in 7 out of 29 samples (mean 5-carbon perfluoropentanoic acid (PFPeA), 6-carbon compound perfluorohexanoaic acid (PFHxA), and 9-carbon perfluorononanoic acid (PFNA) concentration 0.167, 0.261, and 0.100 μg/kg, respectively), toxic metals in 28 out of 29 samples (mean Pb, Cd, As concentration 140.42 ± 23.79, 27.06 ± 4.73, and 69 ± 11.85 μg/kg, respectively), while BPA (mean concentration 1.29 ± 0.19 μg/kg) and MPs [fibers (65%) and fragments (35%)] were detected in all samples. The lack of maximum residual limits and the chronic dietary exposure to complex contaminant mixtures may pose health risks to cats due to cumulative and synergistic effects. These findings underscore the need for continuous, stricter monitoring of contaminants and their residues in raw materials, as well as for a comprehensive risk assessment to ensure pet food safety.

## 1 Introduction

As the demand for companion animals rises and pets are increasingly seen as family members, owners' sensitivity toward their health and wellbeing continues to grow (1). Recent data published by the European Pet Food Industry Federation reported that in 2023, Europe was home to approximately 299 million pets, including 108 million cats and 90 million dogs, and supported an annual production of around 9.1 million tons of pet food, creating a market worth more than €29 billion (2). Italy contributes to the EU pet population with an estimated 65 million companion animals, of which around 9 million are canines and almost 12 million felines (3). Moreover, in 2024, the Italian market's turnover for dog and cat food exceeded 3.1 billion euros, with more than 665 tons of products sold.

Food plays a pivotal role in animal welfare and in the relationship between owners and their pets. Indeed, in addition to fulfilling nutritional needs, it is also perceived by owners as a way to express their love, attachment, and care for their pets (4, 5). Owners are increasingly aware that proper nutrition helps prevent pathologic conditions, such as chronic kidney disease (6, 7) and urolithiasis (8, 9), and promotes an active lifestyle, ultimately improving both their longevity and quality of life (10, 11). As a consequence, they continuously seek the best feed option for their companion animals, trust veterinarians as the primary source of information, and prioritize quality, palatability, ingredients, and freshness when choosing food for their pets (12, 13).

However, pet food can also serve as a vehicle for biological and chemical contaminants that may represent a threat to companion animal health. Although most pet foods are safe, studies have reported the presence microbiological hazards (14, 15), toxic metals (16), antibiotic residues (16–18), pesticides (19), mycotoxins (20), and toxic compound, such as bisphenol A (BPA) (21), melamine and cyanuric acid (22), in commercial pet food. From a One Health perspective, these contaminations also pose a risk to humans, as antibiotic residues and microbiological contamination can contribute to the spread of antimicrobial resistance and pathogenic microorganisms, including *Salmonella, Listeria*, and *Escherichia coli* (23, 24).

Several potential sources of contamination have been hypothesized, including environmental factors, raw ingredients, the manufacturing process, and migration from packaging materials (24, 25).

Toxic metals, for instance, such as lead (Pb), mercury (Hg), arsenic (As), and cadmium (Cd) are widespread environmental pollutants mostly resulting from anthropogenic activities (e.g., metal-based industries, intensive livestock production, use of pesticides, insecticides, and fertilizers) (26). They can accumulate in plants and food-producing animals, whose tissues and by-products are used as ingredients in pet food, thereby contributing to metal contamination in companion animals' diets (16). Raw materials can also contribute to driving other substances, such as antibiotic residues and PFAS (27–30). For instance, experimental data have indicated that bone meal from poultry treated with oxytetracycline (OTC) and slaughtered within the withdrawal period still contained OTC residues, which were responsible for its toxic, pro-inflammatory, and genotoxic effects (27–29). As for PFAS, research by Nomiyama et al. (30) has shown that fish are the primary source of PFAS contamination. However, grain-based pet food contributed non-negligibly to PFAS contamination, whereas meat-based pet food had the lowest PFAS content among the analyzed samples. Beyond the ingredients, the packaging and manufacturing processes can also contribute to pet food contamination, particularly for microplastics (MPs) and BPA (21, 31–33). For instance, research published in 2017 has shown that dogs receiving canned food had increased serum BPA concentrations and altered gut microbiome (34), while MPs were detected in all of the dry pet food samples analyzed by Casella et al. (33).

Chronic consumption of these substances can have serious effects on companion animals' health.

Toxic metals can interfere with antioxidant defense systems, cell signaling pathways, and key cellular processes (growth, proliferation, survival, metabolism, and apoptosis), thereby damaging multiple organs and systems, particularly the kidneys, liver, nervous system, and immune system (35, 36). Antibiotic residues, particularly tetracyclines, have been associated with severe cutaneous and gastrointestinal adverse reactions in both dogs and cats, including exfoliative dermatitis, otitis externa, and diarrhea (17, 37). BPA is a well-known endocrine disruptor that binds to estrogen, androgen, and thyroid hormone receptors, leading to compromised growth and reproductive, nervous, metabolic, and immune dysfunction (38). Exposure to PFAs has been associated with pathologic conditions, such as dyslipidemia, impaired immune response, chronic kidney disease, impaired thyroid function, reproductive issues, autoimmune diseases, and carcinogenicity, particularly in Leydig cells, pancreas, and liver, in humans, domestic, and wild animals (39–42). Finally, MPs' toxicity has been well documented in humans and animals (43), resulting in intestinal, hepatic, and reproductive issues, as well as disorders of the gills and nervous system, and reduced post-hatching survival in fish (44).

Cats are obligate carnivores whose diet in the wild is based on small animal prey, including mammals and birds (45). As a consequence, compared to other animals (e.g., dogs), they have a higher requirement for proteins and specific amino acids (e.g., methionine, arginine, cysteine) (45, 46). Moreover, due to enzymatic deficiencies characteristic of this species, they rely on food as the main source for arachidonic acid, taurine, and vitamin A (46). Dry pet food is widely used in feline nutrition, as it is formulated to meet the nutritional requirements of pets and is appreciated by owners for its affordability, ease of use, and storage (47, 48). Hence, its safety is pivotal to ensure pets' health. However, despite increasing worldwide evidence has revealed the presence of different classes of contaminants in canine and feline blood, as well as in their feed (21, 34, 37, 49–53), most studies focused on a single class rather than on more (21, 30–34, 37, 49).

Therefore, we decided to investigate for the first time the presence of multiple contaminants in a single type of diet, i.e., dry cat food, and, at the same time, to assess the presence of tetracycline residues following the entry into force of Regulation (EU) 2019/6 in light of previous research conducted before such a Regulation.

## 2 Materials and methods

### 2.1 Samples

Twenty-nine, chicken-based cat kibbles from different producers were purchased from the Italian market and used for the chemical analyses. The samples were collected between September and December 2025 at pet stores in central Italy. Three 20 g aliquots were collected from each sample and stored in 50 ml Falcon vials sealed with parafilm. Two aliquots were stored at room temperature, while one was stored at −20 °C until use.

### 2.2 Chemicals and reagents

Certified antibiotic standards used for calibration and quality control in Liquid Chromatography – Tandem Mass Spectrometry (LC-MS/MS) analysis were purchased from Dr. Ehrenstorfer (Augsburg, Germany). Suprapure single-element stock solutions (1,000 mg/kg) for toxic metals (Pb, Cd, Hg, As) and heavy metals standards for ICP-MS calibration were obtained from CPA (Ankara, Türkiye). All analytical-grade solid and liquid reagents, including ethylendiaminetetraacetic acid (EDTA), ammonium formate, methanol, acetonitrile, n-hexane, formic acid, nitrogen gas, argon gas, hydrogen peroxide (H_2_O_2_), and nitric acid (HNO_3_, 65% and 37% suprapure) were supplied by Merck (Milan, Italy).

Pure PFAS standard solutions, including the 13C-labeled standards perfluoro-n-(1,2-13C2)octanoic acid (13C2-PFOA) and sodium perfluoro-1-(1,2,3,4-13C4)octanesulfonate (13C4-PFOS), were obtained from Wellington Laboratories (Guelph, Ontario, Canada). Acetonitrile, methanol, and formic acid used for sample preparation and cleanup were purchased from Merck (Milan, Italy), while ammonium acetate and methanol of LC-MS grade for analysis were purchased from Sigma-Aldrich (Milan, Italy). Oasis WAX SPE cartridges (6 cc, 150 mg) were obtained from Waters (Milford, MA, USA). Ultrapure water was freshly produced daily using a Sartorius system (Milan, Italy).

### 2.3 Antibiotics and coccidiostats analysis

The presence of antibiotic residues in kibbles was investigated using LC-MS/MS (LOQ 50 μg/kg) as previously described (54) with minor modifications. A total of 48 compounds, representing a broad range of pharmacological classes, were screened. The analytes belonged to several classes, including β-lactams (amoxicillin, ampicillin, cefalexin, ceftiofur, cloxacillin sodium salt, dicloxacillin sodium, nafcilin, oxacillin sodium salt, penicillin G potassium), amphenicols (chloramphenicol, florfenicol, thiamphenicol), quinolones (danofloxacin mesylate, difloxacin, enrofloxacin, flumequine, marbofloxacin, nalidixic acid, norfloxacin, sarafloxacin), anticoccidials (diclazuril, maduramycin amonium salt, monensin, nicarbazin, robenidine HCl), macrolydes (erithromycin A, erithromycin B, roxythromycin, spiramycin, tilmicosin, tylosin), lincosamydes (lincomycin), rifamycins (rifaximin), sulphonamides (sulfadiazine, sulfadimethoxine, sulfadoxine, sulfamerazine, sulfameter sodium salt, sulfamethazine, sulfamethiazol, sulfamethoxazole, sulfapyridine, sulfathiazole, sulfachinoxanil, sulfachloropyridazine, sulfachlorpyrazine), and diaminopyrimidines (trimethoprim).

To perform the analysis, 1 g of homogenized kibbles was extracted by adding 2 mL of 1% (w/v) EDTA solution, vortex-mixing for 30 s, then adding 2 mL of acetonitrile and mixing for a further 30 s. Subsequently, 2 mL of methanol was added to the solution, followed by an additional 30 s of mixing. The tubes were placed in an ultrasonic bath at 60 °C for 20 min, then cooled to room temperature. To promote protein and fat separation, the extracts were centrifuged at 4,000 rpm for 10 min, frozen at −20 °C for 12 h, and centrifuged again as before. The supernatant was collected and transferred to a clean tube, and 5 mL of hexane was added for defatting. The mixture was vortex-mixed for 1 min, then centrifuged at 4,000 rpm for 10 min.

The hexane phase was then discarded, and the remaining phase was evaporated to dryness under nitrogen at 45°C. The residue was reconstituted in 1 mL of recovery solution (0.05% formic acid (v/v) in methanol (MeOH)/water, 25:75) and transferred to vials for LC analysis.

Chromatographic separation was achieved on an Ultimate 3000 System (Thermo Fisher Scientific, Waltham, Massachusetts, USA) equipped with an Inertsil ODS-4 column (GL Sciences, Torrance, CA, USA; 2.1 x 50 mm, 3 μm) maintained at 30°C. The mobile phase consisted of (A) water containing 0.1% formic acid and 4 mM ammonium formate, and (B) methanol containing 0.01% formic acid and 4 mM ammonium formate, with a flow rate of 0.05 mL/min, according to a gradient program optimized for the aforementioned multi-class antibiotics.

The LC system was coupled to a Thermo TSQ Quantiva triple-quadrupole mass spectrometer (Thermo Fisher Scientific, Waltham, Massachusetts, USA) operating in heated electrospray ionization (H-ESI) mode. The following source parameters were set: spray voltage 3,500 V (positive ion) and 2,500 V (negative ion); sheath gas 50 arb; auxiliary gas 10 arb; ion transfer tube temperature 320°C; and vaporizer temperature 300°C. Data were acquired in selected-reaction monitoring (SRM) mode over an 11-min run. Compound-specific precursor/product ion transitions, collision energies, and RF lens voltages as listed in the SRM table. The internal calibration was performed using multi-analyte standards (Chebios S.r.l., Rome, Italy). Each 5-point calibration series was considered acceptable if the coefficient of determination (R2) was ≥0.995. The performance of the instrument and the matrix effect were assessed using recovery experiments in which kibbles spiked at representative concentration levels were processed in parallel with the tested samples. The quantification of the individual antibiotics in the kibble samples was based on the corresponding calibration curve, corrected for dilution factors, and expressed as μg/kg of product. The analyses were performed in triplicate, and the results were expressed as the mean (*n* = 3) ± standard deviation (SD) (55).

### 2.4 Tetracyclines

The presence of chlortetracycline, demeclocycline, oxytetracycline, tetracycline, and doxycycline in the kibble samples was investigated using LC-MS/MS (LOQ = 50 μg/kg).

For extraction, 2 g of kibbles were weighed and treated with two consecutive 40 mL aliquots of freshly prepared McIlvaine buffer (pH 4) containing ethylenediaminetetraacetic acid disodium salt and ascorbic acid at 1,000 mg/L.

To obtain the calibration solutions, tetracycline hydrochloride, 4-epi-tetracycline hydrochloride, d8-ciprofloxacin, d8-sarafloxacin, and d5-enrofloxacin were diluted with a solution of acetonitrile (ACN): 0,1% trichloroacetic acid and 0.1% ascorbic acid (70:30), with concentrations from 0 to 50 μg/L for each standard (linear regression *y* = *ax* + *b*). Then, 1 mL of a standard mixture at 500 μg/L was added to each sample, the mixture was brought to a final volume of 200 mL, and then purified by SPE (6 mL−200 mg, Oasis HLB, Waters SpA, Sesto San Giovanni, Italy). Following the purification step, the extract was concentrated to dryness under a nitrogen stream, and 1.5 mL of methyl cyanide was added prior to LC-MS analysis.

The chromatographic analysis was performed using a 1290 Infinity II LC System (Agilent Technologies Italia SpA, Milan, Italy) coupled to an AB Sciex API 6500 mass spectrometer equipped with a heated electrospray ionization (ESI) source. The chromatographic column was an ACQUITY UPLC HSS T3 column (1.8 μm; 150 mm × 2.1 mm, Waters, Milan, Italy) with an injection volume of 2 μL. The mobile phases used were (A) 0.1% formic acid in a 5% ACN solution (v/v) and (B) 0.1% formic acid in ACN (v/v). The column temperature was set to 40 °C, and the flow rate to 0.4 mL/min. The elution began with 1% solvent B from 0 to 1.5 min. Solvent B was then gradually increased to 41% over 1.5–11.5 min. From 11.5 to 12.5 min, solvent B increased to 51%, and to 71% by 14.5 min. A further increase brought solvent B to 99% between 14.6 and 15.1 min. The flow rate was then increased to 0.8 mL/min between 15.6 and 16.1 min, with solvent B held at 99%. Solvent B was then returned to 1% between 16.2 and 16.25 min, and the flow rate was reduced back to 0.4 mL/min from 16.5 to 17.5 min.

The detector was operated in positive MS/MS mode using SRM. Collision gas was set to 10 psi, curtain gas to 30 psi, ion source gases 1 and 2 at 50 and 200 psi, respectively. The ion spray voltage was 5,500 V, and the temperature was maintained at 400°C.

### 2.5 Toxic metals and pesticides analysis

The presence of Pb, Cd, Hg, and As in the kibble was analyzed by Inductively Coupled Plasma – Mass Spectrometry (ICP-MS; Thermo Fisher Scientific, Istanbul, Türkiye) according to the Nordic-Baltic Committee on Food Analysis (NMKL) protocol number 186 (2007), with minor adaptations (Pb, Cd, and As LOQ = 20 μg/kg, Hg LOQ = 40 μg/kg). Briefly, approximately 0.2–0.5 g of homogenized kibble samples were weighed into Teflon vessels suitable for microwave ingestion. A mixture of 9 mL Suprapur^®^ nitric acid (65%, roughly 37%) and 1 mL of H_2_O_2_ was added, and the samples were left uncapped in a fume hood for at least 5 min to allow the acid mixture to penetrate.

The vessels were then sealed and digested in an EthosTM Easy microwave system (Milestone Srl, Sorisole, Italy). According to the protocol, the temperature was gradually increased from 40 to 180°C for 15 min at 1,800W, then held at 180°C for a further 25 min at 1,800W. Then, the samples were cooled from 180 to 40°C for 20 min. Once the digestion was completed, the samples were diluted with ultrapure water to a final volume of 15 mL.

Elemental analysis was carried out using an iCAP RQ System (Fisher, Scientific, Waltham, Massachusetts, USA). Argon (99.9%) was used as plasma gas, in kinetic energy discrimination mode, high resolution, dwell time 0.01 s, one channel, and mass spacing 0.01 u.

Calibration standards for each toxic metal were prepared by appropriately diluting single-element stock solutions (1,000 mg/Kg) in 0.05% nitric acid. The calibration dilution series was considered acceptable when the coefficient of determination (R2) was ≥0.995. A standard blank (0.05% HNO_3_) and a daily reagent blank, prepared by digesting 4 mL ultrapure water with 5 mL HNO_3_ and 1 mL H_2_O_2_, were included in each run to monitor background contamination and instrumental drift.

Before injecting the samples, instrumental stability was verified by injecting the standards and assessing instrument reliability. Metal concentrations in the samples were automatically calculated by the instrument (mg/L) using the calibration curve, and then converted to mg/kg of kibbles according to the following equation:


$$\begin{array}{l} Result\left(mg/Kg\right)=\frac{\left(N\times Vf\right)}{\left(m\times 1,000\right)} \end{array}$$

Where *N* is the amount of metal detected by the device (μg/L), *Vf* is the final volume (mL) of the digested samples, and m is the amount of analyzed sample (g). Additionally, for As, Cd, Hg, and Pb, results were expressed on a dry-matter basis, assuming that the feed moisture corresponded to 12% using the following correction:


$$\begin{array}{l} Metal\;in\;dry\;matter\;\left(mg/Kg\right)=\frac{\left[Value\;from\;ICP\;\left(mg/Kg\right)\times 88\right]}{\left(100-moisture\;\%\right)} \end{array}$$

The analyses were performed in triplicate, and the results were expressed as the mean (*n* = 3) ± SD (55).

On the other hand, the presence of pesticides was carried out using Gas Chromatography-Tandem Mass Spectrometry (LOQ = 10 μg/kg) according to the QuEChERS method (56).

### 2.6 BPA analysis

The concentration of BPA in each sample was determined using a commercial ELISA kit (Boster Biological Technology, Pleasanton, USA) according to the manufacturer's instructions (Sensitivity 10 pg/ml). Briefly, a six-point calibration curve was prepared by serially diluting the BPA standard (2 μL) to final concentrations of 1,000,000, 100,000, 10,000, 1,000, 100, and 10 pg/mL.

For the BPA extraction, 6.5 g of pulverized kibble was mixed with 10 mL of High-Performance Liquid Chromatography (HPLC)-grade water, vortexed for 30 s, and shaken for 1 h on a platform rocker. The suspension was then sonicated for 20 s at RT at 37 KHz and 80% power (Elmasonic Select 30, Elma Schmidbauer GmbH, Singen, Germany), and vortexed for a further 30 s. An aliquot of 2 mL was transferred to a new tube, mixed with 3 mL of ethyl acetate, and centrifuged at 3,000 g for 5 m (Universal R, Bioinzeta, Pomezia, Italy).

Subsequently, 1 mL of the ethyl acetate layer was transferred and evaporated to dryness in a speed vacuum concentrator (Concentrator 5301, Eppendorf, Hamburg, Germany) for 45 min. The dried extract was resuspended in 20 μL of absolute ethanol (100%), vortexed, and diluted with 1 mL of 1X sample dilution buffer. Finally, 100 μL of the diluted extract was added to each well of the ELISA plate. The ELISA assay was conducted according to the manufacturer's instructions, and the absorbance was read at 450 nm. Each sample was analyzed in triplicate.

### 2.7 PFAS analysis

The presence of eleven different PFASs was determined by LC–MS/MS. The target analytes included perfluorobutanoic acid (PFBA), perfluorobutanesulfonic acid (PFBS), perfluoropentanoic acid (PFPeA), perfluorohexanoic acid (PFHxA), perfluorohexanesulfonic acid (PFHxS), perfluoroheptanoic acid (PFHpA), perfluorooctanoic acid (PFOA), perfluorooctanesulfonic acid (PFOS), perfluorononanoic acid (PFNA), perfluorodecanoic acid (PFDA), and hexafluoropropylene oxide dimer acid.

Sample extraction was performed following a protocol developed by modifying previously published PFAS extraction methods (57). The sample pre-treatment and purification procedures are reported below.

Briefly, 2 g of kibble samples, previously ground to a fine powder, were weighed and transferred into 15 mL polypropylene centrifuge tubes. Five milliliters of ACN were added, followed by the internal standards (IS). Then, 50 μL of spiking solution and pure methanol were added to calibrators and unknown samples, respectively. Subsequently, 2 mL of 200 mM sodium hydroxide was added. Samples were vortex-mixed for at least 1 min and then sonicated in an ultrasonic bath for 15 min to enhance analyte extraction. After sonication, 150 μL of 4 M hydrochloric acid (HCl) was added to neutralize the alkaline medium, and samples were vortex-mixed for 30 s. The extracts were then centrifuged at 7,500 × g and 20°C for 15 min. An aliquot of 4 mL of the resulting supernatant was transferred to clean polypropylene tubes for solid-phase extraction (SPE). Sample clean-up was performed using Oasis PFAS SPE cartridges (GCB 50 mg/WAX 200 mg, 60 μm; Waters, Milford, MA, USA). The cartridges were conditioned sequentially with 4 mL of MeOH and 4 mL of ultrapure water. After sample loading, the cartridges were washed with 4 mL of ultrapure water containing 2% formic acid, followed by 4 mL of MeOH. Analytes were eluted with 3 mL of 1% ammonium hydroxide in MeOH. The eluates were evaporated to dryness under a gentle nitrogen stream at 45°C. The residues were reconstituted in 500 μL of a water/methanol mixture (80:20, v/v), filtered through 0.22 μm nylon syringe filters, and transferred into polypropylene vials prior to LC–MS/MS analysis.

Details regarding the LC–MS/MS instrumentation and the analytical conditions used for the separation and quantification of the target compounds are described by Bardhi et al. (58).

The validated method showed good linearity over the concentration range of 0.005 μg/kg for all analytes (*r*^2^ ≥ 0.99). Accuracy and precision, evaluated at four QC levels (0.01, 0.02, 1.0, and 5.0 μg/kg), were within ±15% and <15%, respectively. The limit of detection (LOD) and lower limit of quantification (LLOQ) were 0.002 and 0.005 μg/kg, respectively. Recoveries ranged from 98% to 106%.

Method specificity was confirmed by the absence of interfering signals at the retention times of the target analytes in blank samples. The use of isotopically labeled internal standards minimized matrix effects, ensuring robust and reliable quantification.

### 2.8 MPs extraction protocol

MPs were extracted from dry food (kibbles) following the optimized protocols described in Muhib et al. (59) and Sridhar et al. (60), with slight modifications. As reported, feed is a complex organic matrix and requires both density separation and chemical digestion. Briefly, 10 g of dry pet food was weighed for each sample. Kibbles were finely minced/ground to obtain a homogeneous powder. The powder was spread in a clean glass container and dried overnight at 40 °C to remove residual moisture. Density separation was performed using a sodium chloride (NaCl) solution with a density of 1.2 g cm^−3^, freshly prepared for each extraction. The dried powder was fully suspended in the NaCl solution and thoroughly mixed to ensure proper dispersion and particle detachment. The suspension was allowed to settle for 60 min, with gentle manual mixing every 10 min.

After sedimentation, the supernatant containing floating MPs was carefully aspirated. The density separation step was repeated three times for each sample, and the three recovered supernatant fractions were pooled. The combined supernatants were vacuum-filtered through a 5 μm polytetrafluoroethylene membrane (PTFE) membrane (Fluoropore^®^) using a glass filtration apparatus. The filtration vessel was rinsed with a small volume of the same NaCl solution to recover particles adhering to the glass surfaces, and the rinse was filtered onto the same membrane. On-filter digestion was carried out to remove residual organic matter. The membrane filter was placed in a clean glass Petri dish and fully covered with 30% H_2_O_2_ to remove residual organic matter. Digestion was performed at 37°C for 2 h. After digestion, the filter was recovered and examined under a stereomicroscope. The maximum inspection time was 20 min per filter. Microplastic particles retained on the filters were identified by visual sorting under a stereomicroscope (Carl Zeiss Stemi™, Oberkochen, Germany) equipped with a USB digital camera, based on morphological criteria, including shape (fiber, fragment, pellet, sheet), color, and size. MPs were classified and measured by Optika ProView software (Optika, Italy). To prevent accidental external contamination, all materials and equipment used throughout the extraction and analytical procedures were thoroughly rinsed with filtered deionized water and allowed to dry prior to use. Furthermore, all sample processing steps were carried out under a clean laminar airflow cabinet to minimize airborne contamination, particularly from synthetic fibers, which are recognized as a major potential source of microplastic contamination.

### 2.9 MPs verification using FTIR and SEM

To perform the physico-chemical characterization of a randomly selected subset of suspected MPs (20%), Fourier Transform Infrared (FTIR) spectroscopy was employed (61–63). However, when particles were too small for accurate analysis, complementary techniques such as Raman spectroscopy were used. In this study, Raman spectra were acquired using an Olympus confocal microscope coupled to a Horiba iHR320 spectrometer. A green laser with a wavelength of 532 nm served as the excitation source, and the incident power on the sample was approximately 4 mW, with a beam diameter of about 5 μm. Material identification was conducted using Spectragryph software, which utilizes a recognition system based on a database of Raman spectra specific to MPs.

### 2.10 Statistical analysis

Data were analyzed using GraphPad Prism 9 software (GraphPad Software, Inc., La Jolla, CA, USA) and expressed as mean ± standard deviation (SD). A descriptive statistic was applied to all contaminants.

## 3 Results

### 3.1 Toxic metals, BPA, antibiotics, and PFAS detection

The concentrations of toxic metals, antibiotics, BPA, and PFAS detected in each sample are reported in Table 1.

**Table 1:** Concentrations of the analyzed compounds in chicken-based kibbles.

Sample	Toxic metals (μg/kg)			BPA (μg/kg)	Antibiotics (μg/kg)		PFAS (μg/kg)		
	Pb (*n* = 24)	Cd (*n* = 15)	As (*n* = 27)		Chlortetra-cycline and 4-epichlortetra-cycline (sum)	Doxycycline	PFPeA	PFHxA	PFNA
#1	165 ± 28	–	51 ± 8	1.396	–	–	0.153	–	–
#2	126 ± 21	25 ± 4	56 ± 9	1.566	–	–	0.181	0.121	–
#3	139 ± 24	36 ± 6	82 ± 14	1.477	–	–	<LLOQ	<LLOQ	–
#4	178 ± 30	25 ± 4	48 ± 8	1.036	–	–	<LLOQ	<LLOQ	–
#5	74 ± 13	22 ± 4	117 ± 20	1.852	–	–	<LLOQ	<LLOQ	–
#6	84 ± 14	–	90 ± 15	1.535	–	–	<LLOQ	–	<LLOQ
#7	84 ± 14	–	128 ± 21	1.257	–	–	0.175	–	–
#8	66 ± 11	26 ± 4	30 ± 5	1.336	–	–	<LLOQ	–	–
#9	144 ± 25	–	56 ± 9	1.205	–	–	<LLOQ	–	–
#10	120 ± 20	–	111 ± 19	1.216	–	–	<LLOQ	–	<LLOQ
#11	443 ± 75	22 ± 4	61 ± 10	1.264	–	–	<LLOQ	–	–
#12	146 ± 25	23 ± 4	51 ± 8	1.280	79 ± 35	95 ± 42	<LLOQ	–	–
#13	340 ± 58	33 ± 5	49 ± 8	1.271	–	–	<LLOQ	–	–
#14	51 ± 9	–	60 ± 10	1.090	–	–	<LLOQ	–	–
#15	79 ± 13	25 ± 4	35 ± 6	1.144	–	–	<LLOQ	–	–
#16	54 ± 9	–	25 ± 4	1.317	–	68 ± 30	<LLOQ	–	–
#17	–	–	–	1.075	–	–	0.174	0.175	–
#18	37 ± 6	31 ± 5	68 ± 11	1.457	–	–	<LLOQ	–	–
#19	62 ± 11	25 ± 4	77 ± 13	0.906	–	100 ± 44	<LLOQ	–	–
#20	214 ± 36	24 ± 4	49 ± 8	1.435	–	–	<LLOQ	0.132	–
#21	–	–	46 ± 8	1.156	–	–	<LLOQ	–	–
#22	–	–	81 ± 14	1.433	–	–	<LLOQ	–	0.100
#23	–	24 ± 4	–	1.381	–	–	<LLOQ	–	–
#24	–	28 ± 5	68 ± 11	1.363	–	–	<LLOQ	–	–
#25	189 ± 32	37 ± 6	155 ± 26	1.295	–	–	<LLOQ	–	–
#26	294 ± 50	–	33 ± 6	1.338	–	58 ± 26	<LLOQ	–	–
#27	55 ± 9	–	43 ± 7	1.286	–	–	0.206	0.618	–
#28	129 ± 22	–	110 ± 18	1.325	–	–	0.114	–	–
#29	97 ± 16	–	83 ± 14	0.971	–	–	<LLOQ	–	–

(<LLOQ indicates the presence of the analyte at concentrations below the lower limit of quantification, which was equal to 0.05 μg/kg for all PFAS).

All the analyzed samples were free from anticoccidials and pesticides. As for antibiotics, 4 out of 29 samples (14%) were contaminated with tetracycline. Specifically, doxycycline was detected in all of the 4 samples, with concentrations ranging from 58 ± 26 to 100 ± 44 μg/kg, while only 1 sample contained residues of chlortetracycline and 4-epichlortetracycline (79 ± 35 μg/kg). Toxic metal residues were detected in almost all the samples, except for sample #17. Pb was present in 24 out of 29 samples (83%), with concentrations ranging from 37 ± 6 to 443 ± 75 μg/kg, As was present in 27 out of 29 samples (93%), with concentrations ranging from 25 ± 4 to 155 ± 26 μg/kg, and Cd was present in 15 out of 29 samples (52%), with concentrations ranging from 22 ± 4 to 37 ± 6 μg/kg. Mg was not detected in any of the samples. BPA was present in all samples (100%) with a mean concentration of 1.299 ± 3.57 μg/kg, while PFAS were detected only in a few samples, in particular PFPeA in 6 out of 29 samples (21%), with concentrations ranging from 0.114 to 0.206 μg/kg, PFHxA in 4 out of 29 samples (14%), with concentrations ranging from 0.121 to 0.618 μg/kg, and PFNA in 1 out of 29 samples (3%), with a concentration of 0.1 μg/kg.

All of the samples contained more than one contaminant. Notably, BPA was present in all samples, with concentrations ranging from 0.906 (sample #19) to 1.852 (sample #5). Eighteen samples (#3, #4, #5, #6, #8, #9, #10, #11, #13, #14, #15, #17, #18, #21, #23, #24, #25, #29) contained two categories of contaminants, notably toxic metals and BPA, except for sample #17, which contained residues of BPA and PFAS. The remaining 11 (#1, #2, #7, #12, #16, #19, #20, #22, #26, #27, #28) contained three categories of contaminants, notably samples #1, #2, #7, #20, #22, #27, and #28 contained toxic metals, BPA, and PFAS, while samples #12, #16, #19, and #26 contained toxic metals, BPA, and antibiotic residues. No samples contained all 4 categories of contaminants.

### 3.2 Occurrence and abundance of MPs in pet food samples

MPs were detected in most analyzed pet food samples (*n* = 29). A total of 122 particles were identified, corresponding to a mean of 4.21 ± 2.46 particles per sample (i.e., 10 g pet food).

**Figure 1:**
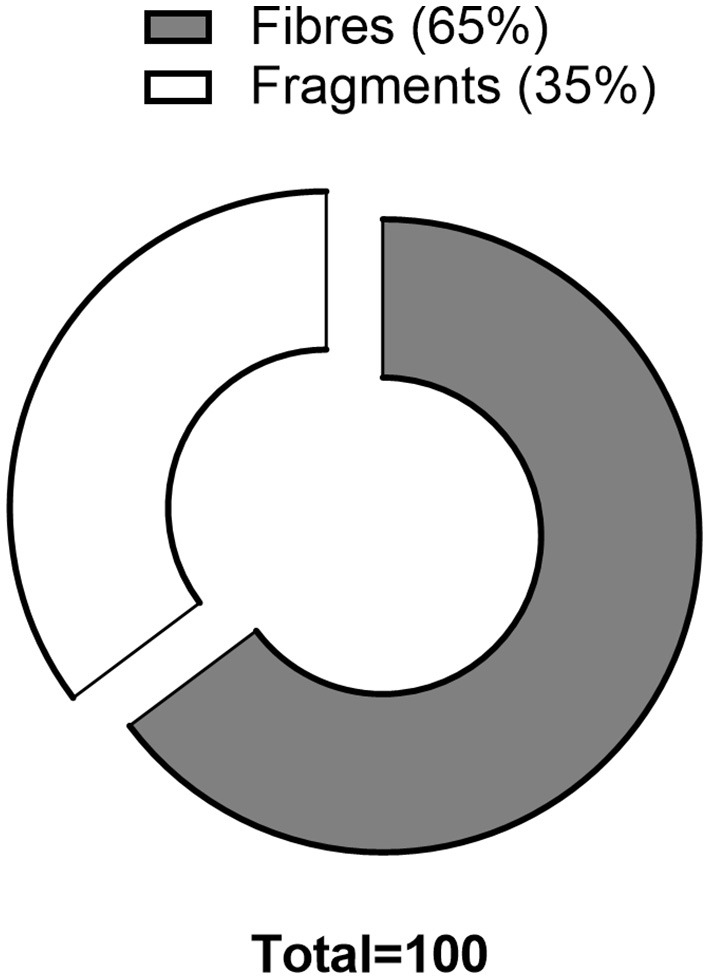
Percentage distribution of MPs by morphotype (fibers vs. fragments) detected in pet food samples (*n* = 29).

Filaments represented the predominant morphotype, accounting for 79 particles (64.8% of total MPs), with a mean of 2.72 ± 1.96 filaments per sample (Figure 1). Fragments were less abundant, totaling 43 particles (35.2%), with a mean of 1.48 ± 1.60 fragments per sample.

#### 3.2.1 Color distribution

**Figure 2:**
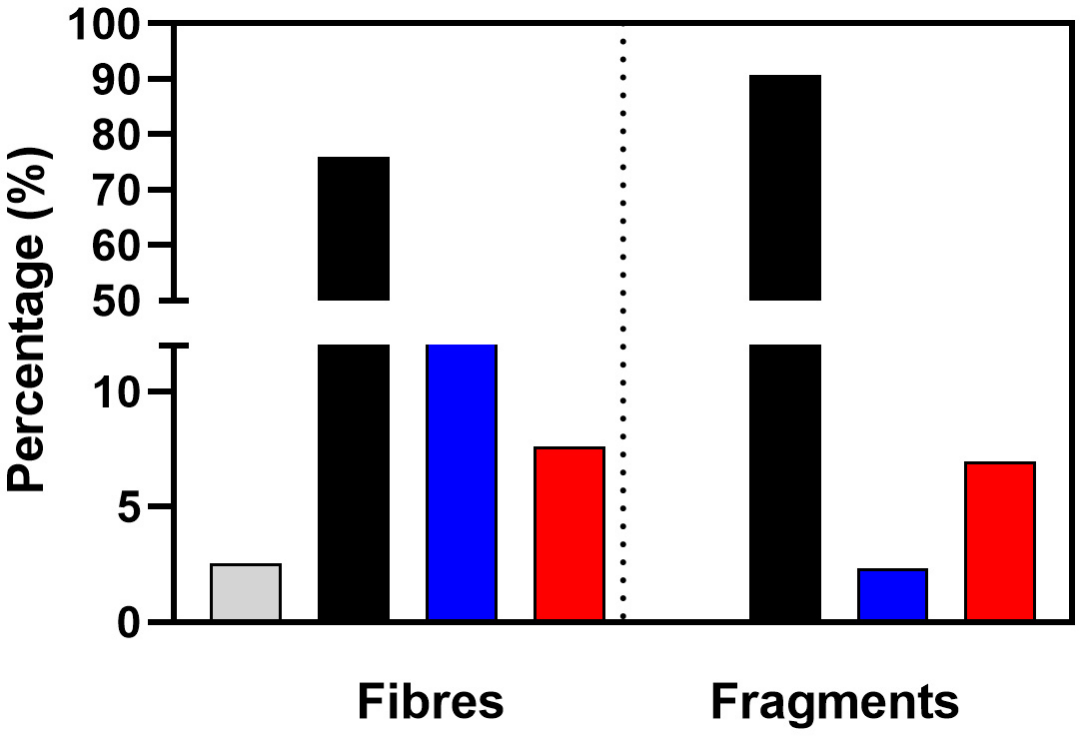
Percentage distribution of MPs colors detected in pet food samples (*n* = 29). Transparent particles are shown in light gray.

Color analysis revealed that black particles were the most frequent in both morphotypes (Figure 2). Among filaments, black fibers accounted for 60 out of 79 particles (75.9%), followed by blue (*n* = 11; 13.9%), red (*n* = 6; 7.6%), and transparent fibers (*n* = 2; 2.5%). Similarly, black fragments predominated among fragments (*n* = 39; 90.7%), whereas blue (*n* = 1; 2.3%) and red fragments (*n* = 3; 7.0%) were less frequently detected. Transparent fragments were not observed.

#### 3.2.2 Size distribution

**Figure 3:**
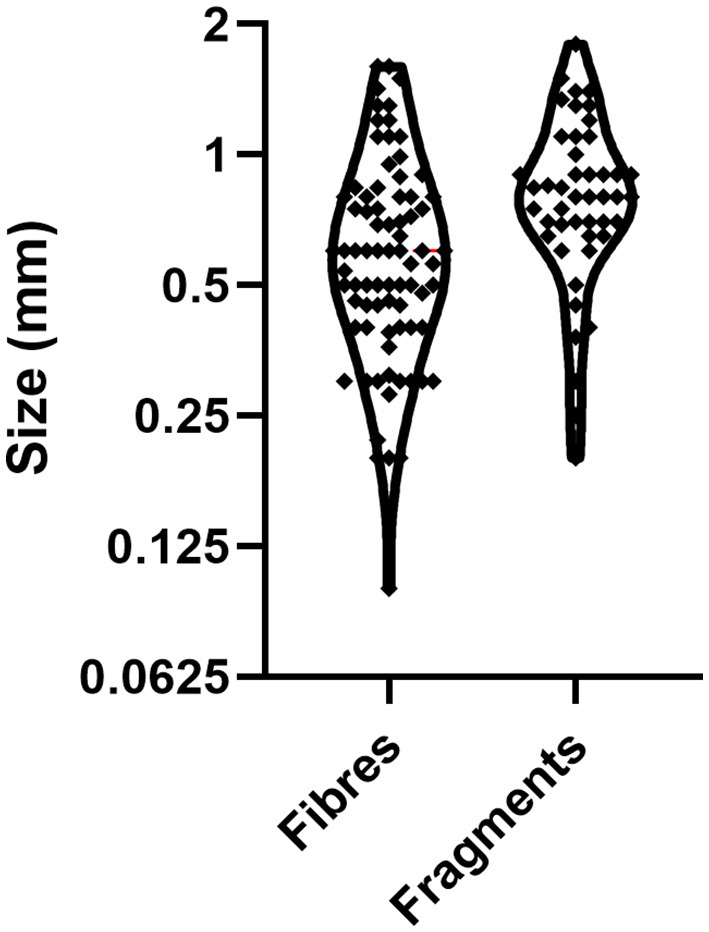
Violin plot showing the size distribution of MPs detected in pet food samples (*n* = 29). Data include all individual particle measurements (range: 0.1–1.8 mm).

Particle size measurements indicated that most MPs were sub-millimetric (Figure 3). The minimum detected size was 0.1 mm, while the maximum reached 1.8 mm. Most particles ranged from approximately 0.2 to 1.2 mm, indicating a prevalence of small secondary MPs, consistent with fragmentation during production, packaging, or environmental exposure.

#### 3.2.3 Chemical characterization of MPs

**Figure 4:**
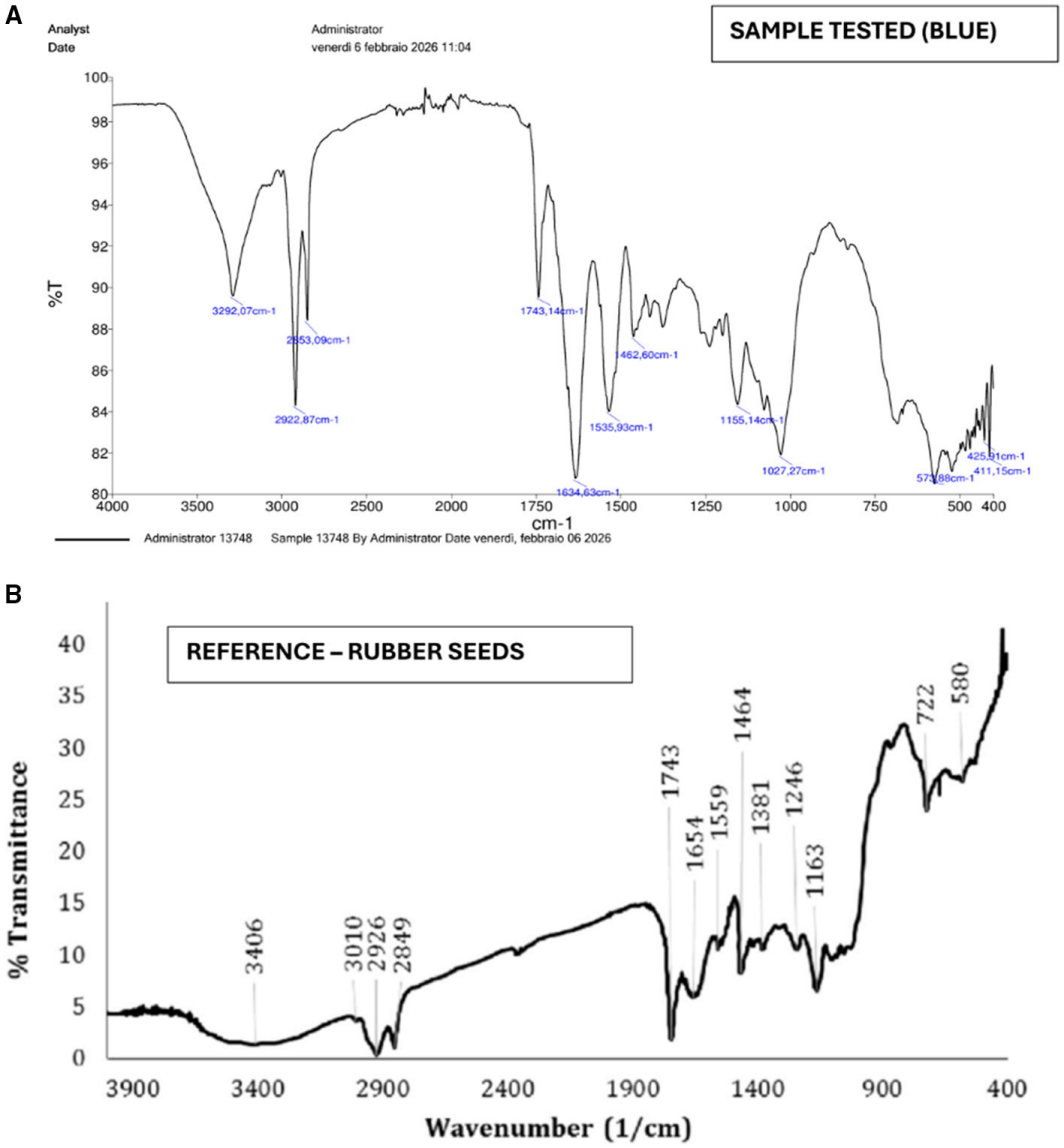
Graphical representation of **(A)** experimental data, and **(B)** spectrum of rubber seeds reported in literature [84].

The physico-chemical characterization of selected particles was performed using Attenuated Total Reflectance - Fourier Transform Infrared (FTIR-ATR) and, when necessary due to their small size, micro-Raman spectroscopy (Figure 4).

Among the analyzed particles, common polymer types such as polyethylene (PE) and polypropylene (PP) were identified based on characteristic vibrational features consistent with widely reported reference spectra. In addition to these conventional packaging-related polymers, one blue particle exhibited an FTIR spectrum characterized by prominent absorption bands at approximately 2,922 cm^−1^ (typically associated with CH, CH2 and CH3 stretching vibrations from aliphatic hydrocarbons), 1,743 cm^−1^ (suggests the presence of carbonyl (C=O) functional groups, which are common in esters or ketones), 1,634 cm^−1^ (corresponds to C=C stretching, indicating the presence of unsaturated bonds, possibly from organic compounds), 1,462 cm^−1^ (generally linked to CH and CH2 bending vibrations), and 1,155 cm^−1^ (indicate C-O stretching, typical for alcohols or ethers) (Figure 4A). The spectrum ranges from 4,000 cm^−1^ to 500 cm^−1^, highlighting a variety of functional groups that contribute to the material's overall chemical structure. When comparing this FTIR spectrum to that of rubber seeds, several distinctions can be noted (Figure 4B). Rubber seeds often exhibit prominent peaks corresponding to specific functional groups, such as C=C bonds, due to the presence of polyisoprene, the main component of natural rubber. This would typically show significant peaks in the region of 1,600–1,700 cm^−1^. In addition, the presence of C=O groups may also be more pronounced in rubber seed spectra due to ester linkages in the rubber matrix. The regions around 2,920 cm^−1^ and 2,840 cm^−1^ are typically strong in rubber seed spectra, indicating strong aliphatic C-H stretching due to the long hydrocarbon chains in the rubber.

**Figure 5:**
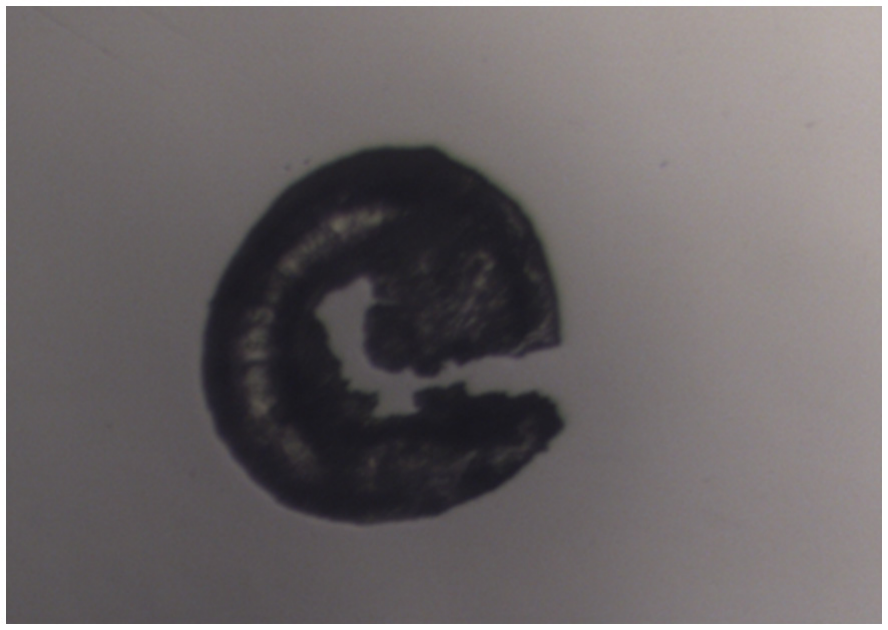
Confocal microscope image of retrieved marine-derived carbonate shell.

On this basis, the particle was tentatively attributed to a natural rubber-based material, potentially originating from nitrile/latex gloves (e.g., blue laboratory gloves). Not all visually suspected particles were confirmed to be synthetic polymers and were therefore excluded from the count. Several samples did not provide consistent FTIR or Raman responses for plastic materials. Spectroscopic analysis indicated that some particles were composed of natural materials, including lignocellulosic fibers and marine-derived carbonate shells. In particular, one sample was described as likely consisting of a marine-derived carbonate shell, rather than a synthetic polymer (Figure 5).

Other particles were also identified as silica-derived materials, further highlighting the importance of spectroscopic confirmation to avoid overestimation of microplastic abundance. Overall, the chemical characterization confirmed the presence of conventional polymers (PE and PP) and a rubber-based particle, likely originating from anthropogenic sources. At the same time, spectroscopic screening allowed the exclusion of non-plastic particles, including natural fibers, biogenic carbonates, and mineral components, thereby increasing the reliability of microplastic identification in pet food samples.

## 4 Discussion

In this study, we shed light on the current state of pet food contamination, particularly in commercially available chicken-based cat kibble. Among the detected contaminants, two antibiotics from the tetracycline family, chlortetracycline and doxycycline, were detected in 4 of 29 samples, with mean concentrations of 79 ± 35 μg/kg and 80.25 ± 35.5 μg/kg, respectively. Tetracyclines, although regulated under Regulation (EU) 37/2010 for foodstuffs of animal origin intended for human consumption (64), have recently been included among the antimicrobials banned in food-producing animals for prophylactic and growth-promoting purposes under Regulation (EU) 2019/6 (65).

Nevertheless, pet food derived from byproducts of food-producing animals is still unregulated for all veterinary drugs, posing a serious concern for the health of companion animals, which are daily exposed to potentially harmful concentrations of antibiotic residues.

For instance, previous *in vitro* (18, 27, 66, 67) and *in vivo* (17, 37) studies have demonstrated the potential toxicity of OTC residues due to the adsorptive capacity of chicken bone fragments, which are typically present in dry pet food formulations at an inclusion percentage of 20–30% (17, 68). In fact, although toxicological studies carried out in dogs showed a low toxicity of oxytetracycline, with a No-Observed-Adverse-Effect-Level (NOAEL) of 250 mg/kg/day (69), tetracyclines hold a high affinity for calcium, which allow them to form stable complexes in bones, which in turn behave like sponges, capable of releasing small amounts of antibiotic depending on environmental pH variations (23, 70).

In this context, Odore et al. (66) first reported a high OTC concentration (1,286.3 ± 256.6 μg/kg) in bone tissue of broiler chickens that received the antibiotic at the dosage and withdrawal times mandated by law for food-producing animals (66, 71). The challenge of a chicken bone extract, achieved by incubating 1 g of bone tissue (1.28 μg/g of OTC) with 10 mL of cell culture medium for 48 h, elicited a significant pro-apoptotic effect on K562 cells and peripheral blood mononuclear cells at 1:1, 1:2, 1:4, and 1:8 dilution ratios (66). Moreover, the 1:4 dilution ratio was shown to significantly induce pro-inflammatory responses, including interferon (IFN)-γ production in CD4+ and CD8+ T lymphocytes and in non-T cells, after 10–12 h of cell exposure (67). It is worth noting that recent insights into food allergy immune regulation have highlighted the importance of IFN-γ in counterbalancing Th2 responses, thereby increasing IL-4 and IgE production and exacerbating allergic sensitization (72). This evidence is more relevant when considering that most cutaneous adverse food reactions in dogs and cats are caused by allergens, including chicken, and can be treated with a dietary switch (73, 74). It is therefore reasonable to hypothesize that tetracyclines can accumulate in chicken bones and, once ingested by pets, trigger cytotoxic and pro-inflammatory phenomena that can synergize with food allergies.

This hypothesis was confirmed by the study of Mazzeranghi et al. (17), who investigated the presence of oxytetracycline in the sera of 18 indoor-housed domestic cats that were used to eat chicken-based kibbles and presented evident clinical symptoms, such as drooling, back and neck intense itching, neck eczema, chronic conjunctivitis, and stomatitis (17). Interestingly, serum oxytetracycline concentration significantly decreased from 108.30 ± 2.88 ng/mL at the first visit to 88.78 ± 4.37 ng/mL after 60 days following the shift to a nutraceutical hypoallergenic diet, and this decrease was accompanied by a significant reduction in pruritus intensity and skin lesion severity score. Although the authors ruled out the presence of an inflammatory condition in skin and ear swabs, they ascribed the skin lesions to pharmacological agents and food allergies (75, 76).

Similar results were also observed by Cerbo Di et al. (37), where 8 dogs that had received chicken-based commercially available kibbles presented clinical manifestations (otitis, diarrhea, generalized anxiety, and dermatitis) and biochemical parameters (alkaline phosphatase, gamma-glutamyl transferase, and urea) alterations, which significantly improved after 15 days of diet shifting. Interestingly, HPLC analysis conducted on 1 g of chicken-based kibbles revealed the presence of OTC at a concentration of 19.25 μg/Kg, while the ELISA test, performed on the dogs' sera to assess the OTC concentration before and after 15 days of diet shifting, highlighted a significant reduction from 0.22 ± 0.12 μg/mL to 0.02 ± 0.03 μg/mL, which accompanied with an improvement in the clinical manifestations.

The presence of tetracyclines in pet food, albeit in only a few samples, raises another concern about antibiotic resistance, as previously reported by Iseppi et al. (77). In fact, the authors reported a higher rate of tetracycline resistance (62.5%) in *E. coli* strains isolated from fecal samples of cats, confirming a possible link between antibiotic-contaminated diet and antibiotic presence in feces.

The analysis of the kibbles also revealed the presence of BPA in all samples, with a mean value of 1.299 ± 3.57 μg/kg, which is in line to that reported by Maršálek et al. (31), who evaluated its concentration in commercial cat dry food (1.18 ± 0.518 ng/g) commonly available in pet shops in the Czech Republic. Although the authors indicated cans as the sources with the highest possibility to release BPA (24.6 ± 34.8 ng/g) also compared to trays (1.58 ± 0.974 ng/g) and pouches (0.591 ± 0.592 ng/g), they ascribed such a difference to the presence of epoxy resins, which are commonly used in cans to prevent the contact between the food and the metal wall (21, 34). Conversely, the low BPA concentrations observed in the other samples were attributed to the lack of an internal coating in dry food, the presence of aluminum in trays, or the use of BPA-free plastic coatings in pouches. Moreover, it was hypothesized that BPA contamination of dry food, trays, and pouches might have occurred during pet food production, whereas in canned food it was a consequence of pH variations (78–81).

Based on literature evidences, our study can be therefore considered one of the few that investigated the presence of BPA in dry pet food, since most of reports focused on canned pet food (from <0.20 to 136 ng/g in cat food and from <0.20 to 208 ng/g in dog food) (21, 34, 82, 83) and/or the serum concentration of cats (1.06 ng/mL) (84) and dogs (1.4 ± 0.14–1.5 ± 0.2 ng/mL) (34). It is noteworthy that the mean amount of BPA we found in our samples is 1,000 times higher than the European Food Safety Authority's (EFSA) tolerable daily intake (TDI) for humans, set at 0.2 ng/kg/day, which is considered capable of impairing the immune system (85). Nevertheless, BPA toxicity extends also to mammary gland and prostate cancer (86), diabetes (87), decreased pituitary luteinizing hormone secretion and Leydig cell testosterone production (88), decreased sperm motility and abnormalities in sperm morphology (89), hyperactivity, placental cells apoptosis and necrosis (90). BPA has also been observed to reduce tyrosine hydroxylase activity in rats (91), thus resulting in hyperactivity, although Kovaríková et al. (84) did not find any association with thyroid function in 69 healthy cats aged ≥7 years. In cats, in particular, no extensive studies have been conducted on BPA toxicity, and more research is needed (92). However, *in vitro* research has observed a negative effect on feline uterine cells' contractility, hypothesizing a potential implication of BPA in queen infertility (93). Furthermore, *in vivo* research has shown that BPA can affect cats' nervous systems, particularly by impairing visual information processing (94, 95). Finally, a possible correlation between BPA exposure and hyperthyroidism has been speculated; however, research by Kovaríková et al. (84) found no correlations between BPA serum levels and thyroid hormones in cats.

However, the authors underlined the significant differences in BPA levels between canned and non-canned food (1.23 ± 0.935 ng/mL vs. 0.774 ± 0.795 ng/mL) that cats were reported to eat. This last observation was also consistent with Edinboro et al. (96), who reported that cats fed canned food are at great risk of developing hyperthyroidism.

Contrary to BPA, several reports revealed high concentrations of toxic metals in dry pet food rather than in canned pet food, with Pb, Cd, and As values being 10 to 1,000 times higher than those observed in this study (Pb, 140.42 μg/Kg; Cd, 27.06 μg/Kg; As, 69 μg/kg), when considering dry cat food (16, 97, 98). For instance, Zafalon et al. reported a Pb and Cd concentration of 11.567 and 2.664 mg/Kg, respectively (16), Semerjian et al. a Pb and Cd concentration of 1.15 and 0.1 mg/Kg, respectively (98), Kalicharan et al. (48) a Pb, Cd and As concentration of 7.24, 3.15, and 2.92 mg/Kg, respectively (48), and Du et al. a Pb, Cd and As concentration of 3.93, 0.142, and 0.16 mg/Kg, respectively (97). It is worth noting that the first three reports showed lower concentrations of toxic metals in poultry-based pet food than in pork-, red meat-, and fish-based ones. On the other hand, studies conducted only on dog dry food showed strong agreement with our data [140.42 vs. 158.42 μg/kg for Pb (53), 69 vs. 54 μg/kg, and 27.06 vs. 15 μg/kg for Cd and As, respectively (50)]. However, the mean Pb value observed by Kim et al. (50) was 10 times lower than ours (140.42 vs. 37 μg/kg). The authors, who compared As, Cd, Pb, and Hg concentrations in dry dog foods with red meat, poultry, and fish as protein sources, confirmed a direct correlation between metal concentrations and protein sources, with poultry showing the lowest levels compared with red meat and fish. Toxic metals may have serious detrimental effects on feline health. Pb, Hg, and As, in particular, have been associated with neurologic and gastrointestinal symptoms, including ataxia, lethargy, vomiting, diarrhea, and weight loss, as well as depression and anorexia (99–102).

According to Directive 2002/32/EC on undesirable substances in animal feed, all detected metals showed concentrations below the maximum allowed content (As, 10 mg/Kg; Cd, 2 mg/Kg; Pb, 5 mg/Kg) (103), thus indicating low exposure concentrations for all considered metals, reducing their accumulation and the risk of adverse effects from chronic consumption.

The analysis of the 29 dry cat food samples revealed a heterogeneous pattern of PFAS contamination (PFPeA, PFHxA, and PFNA mean concentration 0.167, 0.261, and 0.100 μg/kg, respectively), which provides this study with great importance due to the lack of profiles of such compounds in pet food scientific literature (30). In addition, although pet food has been regarded as an important source of PFAS (104) and several reports have highlighted the presence of such compounds in pet sera (105–109), no direct correlation between their presence and pet food consumption has been found. Nomiyama et al. (30), who recently investigated the presence of PFAS in dry (mean value of 0.4 and 1.0 ng/g for dog and cat food, respectively) and wet (mean value of 0.11 and 0.56 ng/g for dog and cat food, respectively) pet food samples, reported the occurrence of great amounts of perfluorotridecanoic acid, perfluoroundecanoic acid, and PFOS (30). Moreover, the authors reported the occurrence of higher concentrations of these substances in fish-based products rather than in meat and a hazard quotient >1 across all food types, thus indicating a higher risk of exceeding the tolerable weekly intake of 4.4 ng/Kb bw (0.63 ng/Kg/day) for major PFAS (PFOA, PFNA, PFHxS, and PFOS) (110). In this sense, our results indicate PFAS levels 10 times lower than those observed in dry cat food, thus limiting the possibility of exceeding the tolerable daily intake when considering the manufacturer's daily ration, but not ruling out a possible bioaccumulative process following chronic intake. In particular, PFPeA was the most frequently detected compound, present in 6 out of 29 samples, whereas PFHxA and PFNA were observed sporadically, suggesting isolated contamination events. In some cases, the co-occurrence of PFPeA and PFHxA may indicate common contamination sources, potentially linked to specific ingredients or production batches rather than a systematic distribution.

The predominance of short-chain PFAS, such as PFPeA and PFHxA, is consistent with the industrial substitution of long-chain PFAS following regulatory restrictions (111). Nevertheless, measurable concentrations of these compounds indicate that replacing long-chain analogs with short-chain PFAS does not fully rule out exposure risks.

Historically, regulated PFAS, including PFOA and PFOS, were either absent or below the limit of quantification, which may provide some reassurance about the safety of pet food with respect to these persistent and toxic compounds.

Variability in PFAS occurrence across brands, product categories, and types (e.g., kitten vs. adult formulas) suggests that contamination is unlikely to be strictly associated with market positioning or product type, but may instead reflect ingredient selection or specific production processes.

As observed in PFAS, scientific literature on pet food contamination by MPs is also scarce. In fact, only Zhang and coworkers (51) have conducted such research so far. However, the authors ascribed the contamination of the feces of cats by polyethylene terephthalate (PET, 61,000 ng/g), polycarbonate (PC, 230 ng/g) and their monomers, terephthalic acid (TPA, 260 ng/g) and BPA (44 ng/g), respectively, to dust and indoor air rather than pet food (PET, 3,800 ng/g; PC, 110 ng/g; TPA, <11 ng/g; BPA, <1.4 ng/g). The presence of PFAS in food is a significant hazard for feline health. Compared to other species, cats have a reduced capacity for xenobiotic metabolism; consequently, they may accumulate PFAS, particularly in the kidneys, thereby increasing the risk of developing chronic kidney disease (30).

Conversely, we observed consistent contamination by low numbers of MPs, predominantly fibers and dark-colored, within the sub-millimetric range. This is relevant because fibers can increase free radical production, compromise the integrity of the intestinal barrier, and disrupt the balance of the gut microbiota, leading to intestinal disturbance and chronic inflammation (112). In cats, particularly, chronic exposure to MPs may result in local fibrosis, chronic enteritis, and increased susceptibility to enteropathies.

The observed contamination pattern aligns with previously reported data on processed foods and packaged products, in which fiber-type MPs are frequently detected and often associated with airborne deposition, handling, or packaging processes (33, 113, 114). Studies on animal feed have demonstrated that feed can represent an important exposure pathway, with variability in abundance and size distribution reflecting multiple contamination routes along the production chain, including raw materials, handling, processing, and packaging (33, 115). In particular, the predominance of fiber-shaped MPs is consistent with their persistence in the environment and their widespread occurrence across different feed matrices.

Similarly, recent evidence in commercial pet food confirms that MPs contamination is ubiquitous and largely driven by a cumulative contribution of raw ingredients, industrial processing (e.g., extrusion and grinding), and migration from packaging materials such as PE, PP, and PET (33). In this context, the predominance of fibers vs. fragments has been linked to different contamination sources, with fibers mainly associated with environmental and airborne inputs, and fragments more related to packaging abrasion and mechanical stress during processing. The high prevalence of colored MPs may be linked to the use of pigmented packaging materials for feed ingredients, which can fragment during grinding (116). Moreover, the predominance of black particles observed in both morphotypes may indicate a contribution from secondary MPs resulting from the degradation of packaging materials or from environmental weathering. Darker particles are commonly associated with aged or highly processed plastic sources, whereas lighter or transparent MPs are more often associated with primary materials or agricultural films (114).

In parallel, methodological reviews emphasize that matrix complexity and extraction protocols can strongly influence detection efficiency and reported abundance, underscoring the need for standardized analytical procedures (33, 117).

## 5 Conclusions

In conclusion, our study assessed for the first time the possible co-occurrence of multiple contaminants in pet food samples. In particular, 29 dry cat food samples were contaminated with one or more contaminants, including toxic metals (Pb, Cd, As), BPA, MPs (PE and PP), PFAS (PFPeA, PFHxA, and PFNA), and antibiotics (chlortetracycline and doxycycline). BPA mean concentration (1.299 ± 0.03 μg/kg) was 1,000 times higher than EFSA's tolerable daily intake for humans; tetracyclines mean concentration (80.25 ± 10.22 μg/kg) was 4 times lower than the NOAEL; toxic metals mean concentrations (Pb = 140.4 ± 20.29 μg/kg, Cd = 27.07 ±1.26 μg/kg, As = 69 ± 6.22 μg/kg) were at levels below the maximum allowed content imposed by Directive 2002/32/EC; and PFAS and MPs mean concentrations (0.19 ± 0.04 μg/kg and 4.21± 2.46 particles per sample, respectively) were far below those reported for similar compounds in other studies. Except for BPA, which warrants further investigation and the setting of maximum limits based on the human TDI, the clinical consequences of chronic consumption of the other contaminants should not be neglected, especially in light of the possible emergence of antibiotic resistance.

## Data Availability

The original contributions presented in the study are included in the article, further inquiries can be directed to the corresponding authors.
